# The microbiome research in obstetrics and gynecology is getting attention—Some reasons why

**DOI:** 10.1111/aogs.70184

**Published:** 2026-03-07

**Authors:** Hana Shabana, Kenny A. Rodriguez‐Wallberg

**Affiliations:** ^1^ Division of Gynecology and Reproduction, Department of Reproductive Medicine Karolinska University Hospital Stockholm Sweden; ^2^ Laboratory of Translational Fertility Preservation, Department of Oncology‐Pathology Karolinska Institutet Stockholm Sweden

Research on the microbiome and its impact in women's health is becoming a hot topic. There is growing evidence that microbial diversity is associated with beneficial health effects in general, and  the increasing number of reports in obstetrics and gynecologic research is noteworthy indicating beneficial effects of specific microbiome features on the  likelihood to achieve pregnancy and the higher chance to maintain maternal and perinatal health, as highlighted recently by the FIGO Reproductive Endocrinology and Infertility Committee.^1^ However, although it is already clear that the microbiome is not a passive bystander, the research field is huge and translation into clinical practice is still limited.

In obstetric health, lessons have been gained since the initial reports of the Human Microbiome Project published in *Nature Medicine*, indicating lower vaginal levels of Lactobacillus in women with preterm births compared to controls, as well as a correlation with pro‐inflammatory cytokines in the vaginal fluid of the preterm cases.^2^ Further on, the recent network meta‐analysis published in *Reproductive Sciences* summarizing the current worldwide literature in the field, 17 studies published between 2014 and 2021, supports that a low‐lactobacilli vaginal microbiome during pregnancy could be predictive of preterm birth.^3^

However, the microbiome is modifiable, in particular by antibiotic drugs, but also by the commonly used medications to reduce gastric acid secretion, such as the proton pump inhibitors (PPIs), which are widely used during pregnancy. Obstetric drug safety has traditionally been defined by the absence of teratogenicity or overt maternal harm; thus, microbiome‐mediated effects remain invisible within this framework. In our Swedish nationwide cohort study published recently in *The Lancet Obstetrics, Gynaecology & Women's Health*, the exposure to PPIs previously or during early pregnancy was associated with a higher risk of maternal infections both during pregnancy and postpartum, those including bacterial, viral, gastrointestinal‐ and respiratory infections, in women that did not have an a‐priori higher risk for infections.^4^ The rationale of the study was based on the vast majority of studies including a meta‐analysis in non‐pregnant individuals indicating a consistent pattern in which PPI exposure is associated with increased risk of infections, most notably for enteric pathogens such as *Salmonella*, *Campylobacter*, and *Clostridioides difficile*,^5^, ^6^ and more heterogeneously for respiratory infections.^7^, ^8^

Maternal infection has a demonstrated negative impact with higher risks for preterm birth, impaired fetal growth, and neonatal morbidity, mediated largely through inflammatory and immune pathways.^9^ A PPI should therefore not be regarded merely as a symptom‐relief drug, but as an ecological modifier in a finely balanced immunological state. Considering obstetric pharmacotherapy in a new biological context, medications act on a dynamic maternal–fetal microbial system, and their safety cannot be fully understood without considering microbiome‐mediated effects. Are we entering a new paradigm in pharmacology? Medications exert not only host‐directed effects but also reproducible alterations of the microbiome, associated with infection risk, immune modulations, and long‐term health, particularly during sensitive windows such as pregnancy. Clinical guidelines should account for this in women prone to infections and may prefer a lower effective dosage and individualized risk–benefit assessment.

In neonatal health research, the recent study published in *Acta Obstetricia et Gynecologica Scandinavica* by Foessleitner et al.,^10^ explored the maternal microbiome during pregnancy and delivery, and also the early development of the neonatal microbiome after cesarean section. Among its main contributions is its time‐based perspective. The microbial colonization of newborns appears to develop over time and from several sources, rather than from a single exposure at birth. These challenges simplified clinical assumptions about the “missing” microbial exposure after cesarean delivery. Earlier assumptions often suggested that the moment of birth, particularly passing through the birth canal, determined neonatal microbiome composition after birth. The study showed that microbiome colonization occurs after birth and develops over time. The widely held belief that cesarean‐delivered newborns are primarily defined by the absence of vaginal exposure has been reformed, revealing instead that their microbiome is shaped by what the newborns are exposed to postnatally, rather than what they did not encounter. Aside from that, the study also showed that despite what we previously assumed about the maternal microbiome becoming increasingly unstable late in pregnancy, healthy pregnancies actually have a stable microbiome, suggesting that instability is a sign of disease rather than normal physiological adaptation. More studies are needed, and with longer follow‐up, to find the impact of the microbiome on long‐term health outcomes.

In reproductive medicine, also microbiome research has captured attention, and microbiome‐targeted interventions are being explored to improve implantation and pregnancy rates, as reviewed in the recent systematic review of Doroftei et al. in *Acta Obstetricia et Gynecologica Scandinavica*.^11^ Still, small populations in the studies currently restrict robust clinical translation.

The FIGO Committee^1^ may be marking the beginning of a new way of thinking, Microbiome‐Integrated Pharmacology, where medications are no longer viewed as acting on the human body alone, but as interventions within a living human–microbial ecosystem.

## Data Availability

Data sharing is not applicable to this article as no datasets were generated or analyzed during the current study.
